# Long-Term Effects of Repeated Social Defeat Stress on Brain Activity during Social Interaction in BALB/c Mice

**DOI:** 10.1523/ENEURO.0068-22.2022

**Published:** 2022-05-03

**Authors:** Hibiki Okamura, Shinnosuke Yasugaki, Haruka Suzuki-Abe, Yoshifumi Arai, Katsuyasu Sakurai, Masashi Yanagisawa, Hotaka Takizawa, Yu Hayashi

**Affiliations:** 1 International Institute for Integrative Sleep Medicine (WPI-IIIS), University of Tsukuba, Tsukuba, Ibaraki 305-8575, Japan; 2 PhD Program in Humanics, School of Integrative and Global Majors, University of Tsukuba, Tsukuba, Ibaraki 305-8575, Japan; 3 Doctoral Program in Biomedical Sciences, Graduate School of Comprehensive Human Sciences, University of Tsukuba, Tsukuba, Ibaraki 305-8575, Japan; 4 Research Fellow of Japan Society for the Promotion of Science, Chiyoda-ku, Tokyo, 102-0083, Japan; 5 Research and Development Center for Precision Medicine, University of Tsukuba, Tsukuba, Ibaraki 305-8575, Japan; 6 Master’s Program in Medical Science, Graduate School of Comprehensive Human Science, University of Tsukuba, Tsukuba, Ibaraki 305-8575, Japan; 7 Faculty of Engineering, Information and Systems, University of Tsukuba, Tsukuba, Ibaraki 305-8575, Japan; 8 Department of Human Health Science, Graduate School of Medicine, Kyoto University, Sakyo-ku, Kyoto, 606-8507, Japan

**Keywords:** c-Fos, stress

## Abstract

Understanding the long-term effects of stress on brain function is crucial for understanding the mechanisms of depression. The BALB/c mouse strain has high susceptibility to stress and is thus an effective model for depression. The long-term effects of repeated social defeat stress (SDS) on BALB/c mice, however, are not clear. Here, we investigated the effects of repeated SDS in male BALB/c mice over the subsequent two weeks. Some defeated mice immediately exhibited social avoidance, whereas anxiety-like behavior was only evident at later periods. Furthermore, defeated mice segregated into two groups based on the level of social avoidance, namely, avoidant and nonavoidant mice. The characteristic of avoidance or nonavoidance in each individual was not fixed over the two weeks. In addition, we developed a semi-automated method for analyzing c-Fos expression in the mouse brain to investigate the effect of repeated SDS on brain activity more than two weeks after the end of the stress exposure. Following social interaction, c-Fos expression was reduced in several brain regions in the defeated mice compared with control mice. The correlation of c-Fos expression among these brain areas, with exception of the medial prefrontal cortex (mPFC) and central amygdala (CeA), was increased in defeated mice, suggesting increased synchrony. Notably, c-Fos expression in the lateral habenula (LHb) was different between mice that exhibited social avoidance from immediately after the repeated SDS and those that exhibited social avoidance only at later periods. These observations provide insight into the long-term effects of social stress on behavior and brain activity.

## Significance Statement

Stress stimuli underlying psychological diseases are frequently of a social nature, and therefore elucidating the effects of social stress in animal models is critical to understanding the underlying mechanisms of these diseases. We investigated how repeated social defeat stress (SDS) affects behavior and brain activity over subsequent weeks in BALB/c mice, a strain exhibiting high susceptibility to stress. Some defeated mice immediately exhibited social avoidance whereas anxiety-like behavior was only evident at later periods. A semi-automated c-Fos extraction and quantitation method developed for mice revealed that brain activity during social interaction differ among socially-avoidant individuals depending on the timing of the emergence of social avoidance.

## Introduction

Depression is a major health problem, with over 300 million people worldwide estimated to suffer from the disease (World Health Organization, 2017). Patients with depression show loss of interest, increased anxiety, hopeless feelings, significant body weight changes, and sleep disorders (for review, see Kanter et al., 2008; Rakofsky and Rapaport, 2018; Steiger and Pawlowski, 2019). Depression is often caused by chronic stress, which can have long-lasting effects. Elucidating how chronic stress leads to long-term alterations in brain functions is critical.

Mice exposed to chronic stress exhibit phenotypes that resemble those of depressed patients, such as anxiety-like behavior, anhedonia, despair-like behavior, and body weight change (Liu et al., 2018; for review, see Yan et al., 2010; Patel et al., 2019). Moreover, the stress-induced phenotypes are alleviated by antidepressants such as tricyclic antidepressants and selective serotonin-reuptake inhibitors (for review, see Czéh et al., 2016). Thus, mice are useful models for understanding how chronic stress leads to long-term changes in brain functions that underlie depression. Several methods exist for applying chronic stress to mice to induce depression-like phenotypes (for review, see Slattery and Cryan, 2017; Wang et al., 2017). One well-established method is the repeated social defeat stress (SDS) protocol. Repeated SDS is thought to mimic social stress, which is a cause of depression in humans. In the repeated SDS protocol, an experimental mouse is subjected to attacks and threats from larger, aggressive mice for 5–10 min daily for 10 consecutive days (Golden et al., 2011). Following repeated SDS, mice exhibit social avoidance to other mice of the same strain as the aggressor mice. Notably, mice that undergo repeated SDS typically segregate into two subpopulations: “susceptible” mice, which exhibit high social avoidance behavior, and “resilient” mice, which exhibit low social avoidance behavior (Berton et al., 2006; Golden et al., 2011; Higashida et al., 2018; for review, see Krishnan and Nestler, 2008). While the behavioral phenotypes immediately after exposure to repeated SDS are well investigated (Krishnan et al., 2007; Higashida et al., 2018; Ishikawa et al., 2021), the long-term effects of repeated SDS are less well understood. Thus, in the present study, we aimed to examine the long-term effects of repeated SDS on behavior and brain activity.

To examine the long-term effects of repeated SDS, we used BALB/c mice, which are often used in the field of immunity (for review, see Atherton et al., 2002; Kanagaratham et al., 2018). Several studies have shown that BALB/c mice are more susceptible to chronic stress compared with C57BL/6 mice. For example, repeated SDS induces social avoidance more drastically in BALB/c mice compared with C57BL/6N mice (Ishikawa et al., 2021), BALB/c mice that undergo chronic restraint stress exhibit increased levels of corticosterone in the hair or serum compared with C57BL/6 mice (Flint and Tinkle, 2001; Tsuchimine et al., 2020), and BALB/c mice exposed to unpredictable chronic mild stress exhibit marked induction of stress-related genes compared with C57BL/6J mice (Malki et al., 2015).

To examine the long-term effects of repeated SDS on brain activity in BALB/c mice, we focused on c-Fos, a marker of neuronal excitation (Hudson, 2018; for review, see Herrera and Robertson, 1996). The expression of c-Fos immediately after the SDS session is well investigated (Tanaka et al., 2012; Nasanbuyan et al., 2018; Numa et al., 2019; Nagai et al., 2020). In such analyses, however, it is difficult to distinguish the psychological effects of stress and the direct effects of physical injury caused by SDS. Moreover, c-Fos expression immediately after SDS reflects both the short-lasting and long-lasting effects. In this study, we focused on c-Fos expression during social interaction two weeks after the end of repeated SDS. In the social interaction session, defeated mice encountered mice of the same strain as the aggressor mice, but with no physical contact.

c-Fos expression analyses is time-consuming, which limits the number of samples or the number of brain regions that can be examined. To overcome these limitations, we developed a semi-automated c-Fos extraction and quantitation method that enabled us to comprehensively examine c-Fos expression levels in various brain areas.

## Materials and Methods

### Animals

All animal experiments were approved by the Institutional Animal Care and Use Committee of the University of Tsukuba, and all experiment procedures were conducted in accordance with the Guidelines for Animal Experiments of the University of Tsukuba. Adult male BALB/cAnNCrlCrlj mice (referred to as BALB/c mice, 10 weeks old, purchased at six weeks of age from Charles River Laboratories Japan) were used in the study. Mice were housed in standard cages and maintained under a controlled environment (23.5 ± 2.0°C, 50.0 ± 10.0% humidity, 12/12 h light/dark cycle, lights on at 9 A.M.). Food and water were available *ad libitum*.

### Repeated SDS

SDS was performed as described previously (Golden et al., 2011; Higashida et al., 2018; Nie et al., 2018) with some modifications. Considering that BALB/c mice exhibit higher stress susceptibility than C57BL/6 mice (Shanks et al., 1991; Razzoli et al., 2011; Tsuchimine et al., 2020; Ishikawa et al., 2021), the stress exposure period was set to 7 d. Before 7 d of SDS, screening for aggressor mice (male ICR, retired from breeding, purchased from Japan SLC) was performed. In the screening, aggression was evaluated by co-housing each mouse with a novel BALB/c mouse for 3 min daily for 3 d. Aggression was evaluated by the latency to attack and the number of attacks. Only ICR mice that showed aggressiveness on both the second and third days were used in subsequent experiments. Before repeated SDS, BALB/c mice were singly housed for one week. Experimental BALB/c mice were then transferred to the home cage of an aggressor ICR mouse for 10-min daily between zeitgeber time (ZT)0 and ZT1. After a 10-min SDS session, mice were returned to their home cage and kept isolated until the SDS session on the next day. To minimize the variability in the interaction of each BALB/c mouse with the aggressor ICR mice, the combinations of experimental BALB/c mice and aggressor ICR mice were altered each day. Considering that prolonged social isolation itself can lead to behavioral changes such as enhancement of anxiety-like behavior (for review, see Pinna, 2019), control mice that were not exposed to repeated SDS were also kept isolated for an equal period. During the SDS sessions, control mice were transferred to a novel cage and allowed to freely explore for 10 min during the same ZT. Body weight was measured before each SDS session. Body weight change (%) was calculated by the following index:

(body weight on day 8 – body weight on day 1)/body weight on day 1 x 100 (%).

### Behavioral analysis

Before the experimental procedure, the mice were handled for 2 min twice daily for 5 d. Mice were transferred to the behavioral testing room and acclimatized to the experimental environment for at least 10 min. The apparatuses were sterilized with weakly acidic water before each test session. All behavioral tests were performed from ZT0 with 40 lux of light at the bottom of the cage.

### Social interaction test (SIT)

The SIT was performed as described previously (Higashida et al., 2018) with some modifications. Briefly, a BALB/c mouse was placed in an open field arena (40 × 40 × 40 cm^3^) in which a novel male ICR mouse was placed in a wire mesh enclosure at one end. The BALB/c mouse was first placed in the corner located opposite to the side where the ICR mouse was placed and allowed to freely explore for 150 s. Before SIT, all BALB/c mice were habituated to the same arena in the absence of the ICR mouse for 150 s (SIT habituation). The behavior was recorded using a video camera located above the center of the arena. The interaction zone was defined as the area occupying 1/3 of the arena area located closest to the side containing the ICR mouse, and the avoidance zone was defined as the area occupying 1/4 of the arena area located furthest from that side. Video data were analyzed with the SMART 3.0 Video Tracking System (PanLab Harvard Apparatus). Avoidant mice and nonavoidant mice were defined as described previously (Higashida et al., 2018), i.e., mice that spent >50% of the time in the avoidance zone during the SIT were defined as avoidant mice, and the remaining mice were defined as nonavoidant mice.

### Elevated plus maze (EPM)

The EPM task was performed as described previously (Ramirez et al., 2015; Ano et al., 2019) with some modifications. The apparatus contains two open arms (30 × 5 cm) with no walls and two closed arms (30 × 5 cm) with 20-cm-high walls. The open and closed arms emanated from a 5 × 5 cm center area with the open and closed arms arranged 90° from each other, such that the maze formed the shape of a plus sign. The arms were elevated 50 cm above the ground. A BALB/c mouse was placed on the center area of the apparatus and allowed to freely explore for 10 min. The behavior was recorded using a video camera from above the center of the maze, and the data were analyzed with the SMART3.0 Video Tracking System.

### c-Fos immunohistochemistry

More than 1 d after the last behavioral test, defeated mice and control mice were exposed to a novel ICR mouse in a manner similar to the SIT; each BALB/c mouse was placed in the open field arena where a novel male ICR mouse was placed in a wire mesh enclosure and allowed to freely explore for 300 s (social interaction). After 2 h, the BALB/c mice were deeply anesthetized and transcardially perfused with 10% sucrose water (w/v) followed by ice-cold 4% paraformaldehyde (w/v) in 0.1 m phosphate buffer (PFA/PB). The brains were removed and postfixed in 4% PFA/PB at 4°C overnight, and then incubated in 30% sucrose (w/v) in PBS at 4°C for 2 nights. The brains were embedded and frozen in OCT compound (Sakura Finetek), and sectioned at 40 μm using a sliding microtome (Yamato Kohki). The brain sections were rinsed with 0.3% H_2_O_2_ for 10 min and washed three times with TBST [TBST tablet (Takara) dissolved with MilliQ water, pH 7.6]. The brain sections were incubated overnight with a primary antibody (1:5000 anti-c-Fos antibody produced in rabbit, F7799, MilliporeSigma) at 4°C. The next day, the sections were washed twice with TBST at room temperature (RT), then incubated with the secondary antibody (Biotin-SP donkey anti-rabbit IgG, AB_2340593, Jackson ImmunoResearch) for 1 h at RT. After washing twice with TBST, the sections were incubated with avidin-biotin complex (VECTASTAIN Elite ABC Standard kit, PK-6100, VECTOR Laboratories) for 1 h at RT. The sections were then washed twice with TBST and incubated with 3,3’-diaminobenzidine (Dojindo, 349-00903). The sections were mounted on a slide glass and dried for at least one night. To dehydrate the sections, the sections were placed for 3 min each in 70% ethanol, 90% ethanol, 100% ethanol (three times), and xylene (three times). Finally, the sections were covered with ENTELLAN NEW (Merck, 1.07961.0100) mounting medium. Images were captured with NanoZoomer-XR (Hamamatsu Photonics), with a resolution of 0.000908 mm/px.

### Semi-automated c-Fos extraction and quantitation

c-Fos signals were detected and quantified in each brain region of the area of interest on the microscopic image using the following three image analysis steps. In step 1, each area of interest was cropped from the microscopic image using the *exclude edge* function in Analyze Particles of ImageJ (National Institutes of Health). The cropped image was then converted to a grayscale image and its background was subtracted using the *rolling ball* plug-in (radius = 50 pixel (px)). The mean filter (kernel size = 5 px), Gaussian filter (σ = 1.3, kernel size = 5 px), and morphologic opening operation (kernel size = 5 px) were then applied to the grayscale image in that order to extract the c-Fos signal candidates with an original image processing program written in Java (Java SE Development kit). If the area of the candidate signal was between 10 and 550 px and its minimum intensity was less than 170, the candidate signal was categorized as a c-Fos signal. In step 2, diagrams from the mouse brain atlas (Franklin and Paxinos, 2007) containing either of the following 19 brain regions of interest [periaqueductal gray (PAG); nucleus accumbens (NAc); central amygdala (CeA); medial prefrontal cortex (mPFC); ventral tegmental area (VTA); cortical amygdala (CoA); lateral septal nucleus ventral part (LSv); bed nucleus of the stria terminalis (BNST); basolateral amygdala (BLA); hippocampal regions CA1, CA2, CA3, and ventral CA1; dentate gyrus (DG); lateral habenula (LHb); medial habenula (MHb); parabrachial nucleus (PBN); locus coeruleus (LC); and the dorsal raphe] were manually transformed based on homography transformation to match the shape of the brain section sample on the microscopic image. This manual transformation was time consuming but insured accurate matching. By overlaying the matched atlas onto the section sample on the microscopic image, each of the 19 brain regions was outlined. In step 3, the number of detected c-Fos signals in each segmented brain region of the microscopic images was divided by the area of the region to calculate the number of c-Fos signals per unit area (c-Fos/mm^2^). The image analysis method was performed on a standard desktop computer with an Intel Core i7-9700T processor and 8-GB installed memory running Microsoft Windows.

### Validation of the semi-automatic c-Fos counting system

To evaluate the sensitivity and precision of the semi-automatic c-Fos extraction and quantitation system, the c-Fos signals on some of the sections were counted manually. First, 10 microscopic images that contained the PAG were randomly selected from the control and defeated groups. Second, the PAG regions were outlined manually using the freehand selection tool in ImageJ. The c-Fos signals in the PAG were then manually counted using the cell counter plug-in in ImageJ under a blind condition in which the investigator did not know the results of the automated analyses. Lastly, sensitivity and precision were calculated by the following definition and index:

For c-Fos counting,

True positive: signals that were categorized as c-Fos signals by both the semi-automatic system and the manual procedure.

False negative: signals that were categorized as c-Fos signals by the manual procedure, but not by the semi-automatic system.

False positive: signals that were categorized as c-Fos signals by the semi-automatic system, but not by the manual procedure.

For area overlap,

True positive: the area that was recognized as PAG by both the semi-automatic system and the manual procedure.

False negative: the area that was recognized as PAG by the manual procedure, but not by the semi-automatic system.

False positive: the area that was recognized as PAG by the semi-automatic system, but not by the manual procedure:

Sensitivity (%) = true positive/(true positive + false negative) x 100,

Precision (%) = true positive/(true positive + false positive) x 100.

### Statistics

Datasets were analyzed using Prism 9 (GraphPad) and Excel (Microsoft) except for the hierarchical cluster analyses. Sample sizes, statistical tests, and *p*-values are indicated in the figure legends. Where applicable, all statistical tests were two-tailed. *P*-values were considered statistically significant when <0.05. Details on sample sizes and results of statistical tests are described in the Extended Data. For hierarchical cluster analysis, each value of c-Fos densities in each brain region of each experimental group was standardized by autoscaling (a conversion of the mean to 0 and the SD to 1 in each region), and hierarchical clustering analyses with the squared Euclidean distance and the Ward’s method were performed using the mean values in each experimental group. The analyses were performed using the *hclust* function within the R Stats Package (all codes and datasets used/generated are available at https://github.com/balb-sds/semi-autmated-cFos-analysis as Extended Data 1). Heatmaps were semi-automatically constructed using Prism 9.

### Code accessibility

The codes described in the paper are freely available online at https://github.com/balb-sds/semi-autmated-cFos-analysis as Extended Data 1.

10.1523/ENEURO.0068-22.2022.ed1 Extended Data 1 A code for Semi-automated c-Fos extraction and quantitation method. Download Extended Data 1, ZIP file.

## Results

### Effects of repeated SDS on BALB/c mice

**Figure 1.**
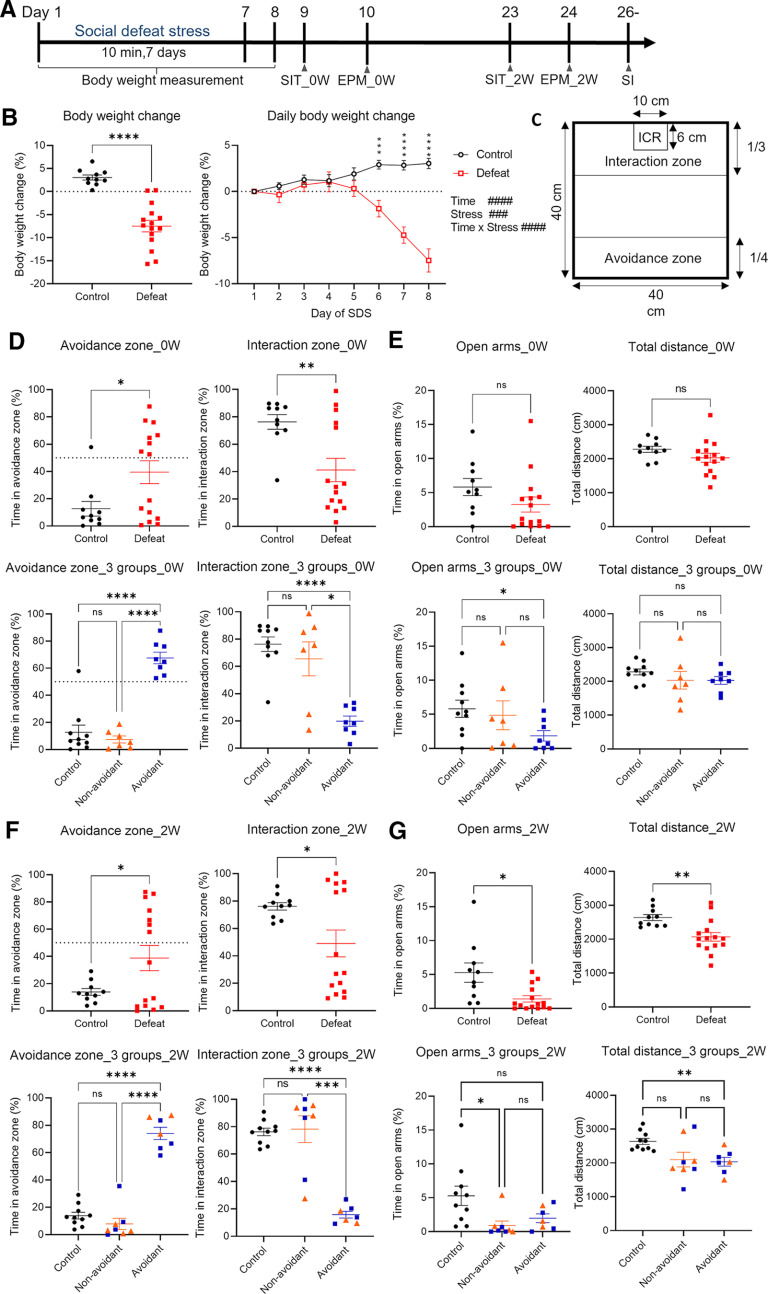
BALB/c mice exhibit altered behavioral phenotypes immediately and two weeks after repeated SDS. ***A***, Experimental timeline of repeated SDS. ***B***, Body weight change on day 8 (left) and daily body weight change during the 7 d of SDS (right). ***C***, Schematic of the SIT arena. ***D***, Comparison of time spent in the avoidance and interaction zones during the SIT_0W between control and defeated mice (top), or among the three groups, stratifying defeated mice into nonavoidant and avoidant mice (bottom). ***E***, Comparison of time spent in the open arms and the total distance during EPM_0W between control and defeated mice (top), or among the three groups (bottom). ***F***, Comparison of time spent in the avoidance and interaction zones during the SIT_2W between control and defeated mice (top), or among the three groups (bottom). ***G***, Comparison of time spent in the open arms and the total distance traveled during EPM_2W between control and defeated mice (top), or compared among the three groups (bottom). Control mice: *n* = 10, defeated mice: *n* = 15, data are presented as means ± SEM. ***B***, ***/###*p* < 0.001, ****/####*p* < 0.0001, Unpaired *t* test for comparison between two groups and two-way repeated measures ANOVA followed by multiple comparison tests with Bonferroni correction for daily body weight change. ***D–G***, ns, not significant, *p < 0.05, **p < 0.01, ***p < 0.001, ****p < 0.0001, Unpaired *t* test with Welch’s correction for comparison between two groups and Welch ANOVA test followed by Games–Howell’s multiple comparisons test for comparison among the three groups. Orange triangles and blue squares indicate nonavoidant mice and avoidant mice in the SIT_0W, respectively. Detailed results of the statistical tests are described in Extended Data Figure 1-1, 2-1, 3-1, 4-1, 6-1.

To elucidate the long-term effects of repeated SDS on BALB/c mice, we exposed BALB/c mice to 7 d of SDS. The experimental timeline is shown in Figure 1*A*. Body weight was decreased in the defeated group from day 6, and further decreased in the following days (Fig. 1*B*). To assess the short-term effects of repeated SDS on behavior, following habituation to the arena for SIT on day 8 (1 d after the last defeat session), we performed the SIT and EPM tasks on day 9 and day 10, respectively (SIT_0W and EPM_0W). The defeated group exhibited higher social avoidance and concomitant lower social interest compared with the control group in the SIT_0W (Fig. 1*D*). Moreover, consistent with previous findings (Higashida et al., 2018), the defeated group could be divided into two subpopulations based on the time spent in the avoidance zone (%), i.e., individuals that spent either more or <50% of the total time in the avoidance zone (Fig. 1*D*). These subpopulations were designated as avoidant mice or nonavoidant mice, respectively. In the EPM_0W, no significant difference was detected between the control and defeated groups or between avoidant mice and nonavoidant mice with regard to time spent in the open arms or in the total distance traveled in the EPM (Fig. 1*E*), suggesting that the anxiety levels of the mice were not increased. To test the long-term effects of repeated SDS on behavior, we performed the SIT and EPM again two weeks after the first series of behavioral tests (SIT_2W and EPM_2W). In C57BL/6J mice, repeated SDS for 10 d causes chronic changes in physiologic states such as sleep that become apparent already 5 d after the end of the stress exposure, and effects of repeated SDS on behavior can last up to four weeks (Berton et al., 2006; Henderson et al., 2017). In the present study, considering that the period of repeated SDS was 7 d, we decided to examine the effects of repeated SDS after two weeks from stress exposure period. Similar to the SIT_0W, in the SIT_2W, the defeated group showed higher social avoidance and concomitant lower social interest compared with the control group, and the defeated group could be divided into avoidant and nonavoidant mice (Fig. 1*F*). The subpopulations of the defeated group in the SIT_2W, however, did not comprise the same individuals as the corresponding subpopulations in the SIT_0W; some individuals moved to the other subpopulation, either from the avoidant to the nonavoidant subpopulation or vice versa (Fig. 1*F*). In the EPM_2W, the time spent in the open arms was reduced and the total distance traveled was decreased in the defeated group compared with the control group (Fig. 1*G*). Thus, the defeated group seemed to develop anxiety-like behavior at some time point during the two-week post-SDS period. The EPM_2W scores did not differ significantly between the avoidant and nonavoidant mice (Fig. 1*G*).

10.1523/ENEURO.0068-22.2022.f1-1 Extended Data Figure 1-1 Detailed results of the statistical analyses in Figure 1. Download Figure 1-1, XLS file.

### Establishment of a semi-automated c-Fos signal extraction and quantitation method

**Figure 2.**
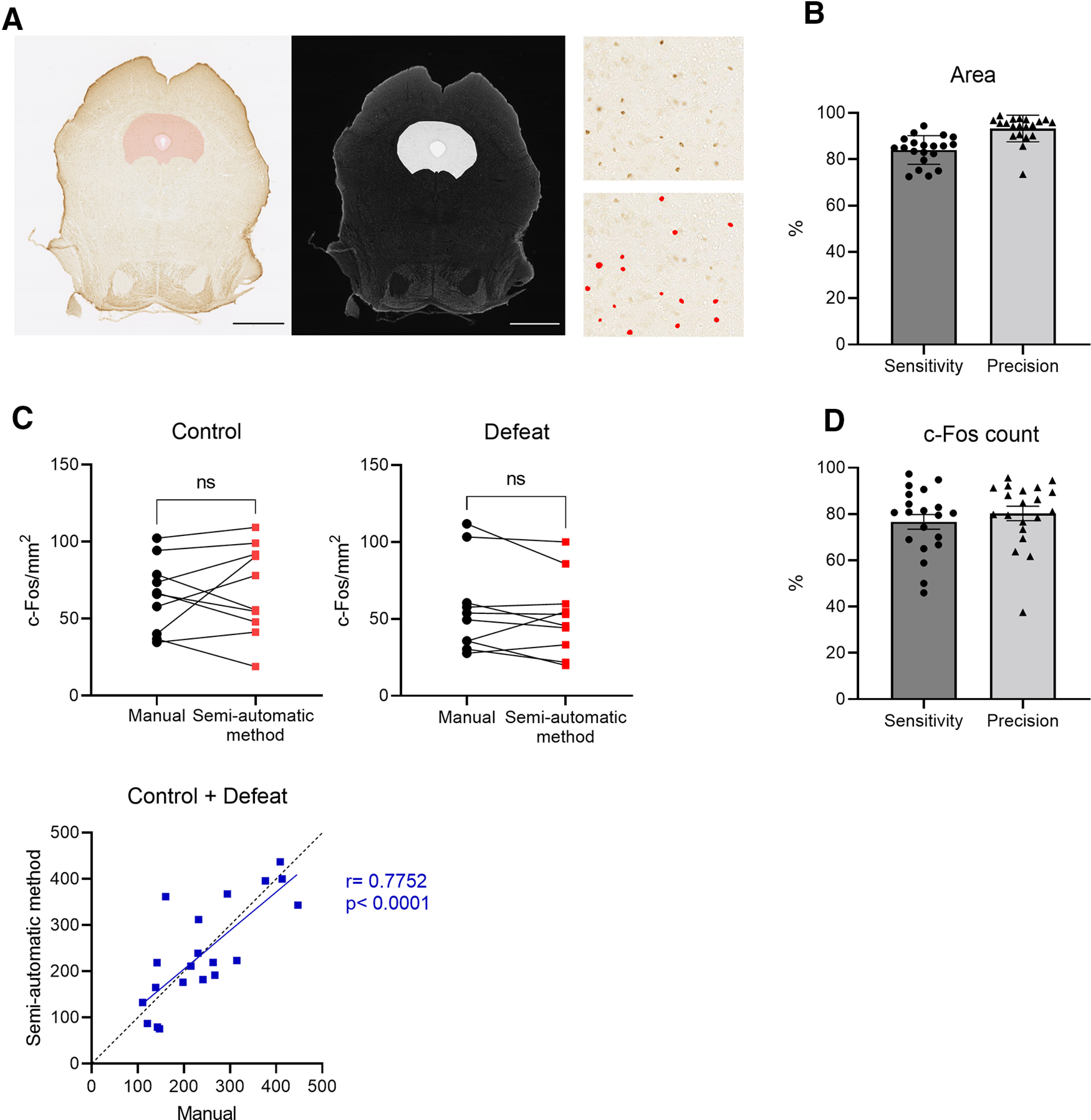
Establishment and evaluation of the semi-automated c-Fos extraction and quantitation method. ***A***, Example of a coronal section that includes the PAG. The pink shadow (left) shows the region outlined as PAG with the semi-automated method (PAG_auto), and the white shadow (middle) shows the PAG region that was manually outlined (PAG_manual). Scale bar: 1 mm. Red dots (right) indicate locations that were detected as c-Fos signals by the semi-automated method. ***B***, The sensitivity and precision for PAG outlining using the semi-automated method. ***C***, Comparison of c-Fos signal densities within PAG quantified either manually or by the semi-automated method, and the scatter plot showing correlations in c-Fos signal densities quantified by the manual and semi-automated methods. ***D***, The sensitivity and precision for c-Fos signal detection within the PAG using the semi-automated method. Control: *n* = 10, defeat: *n* = 10 (10 defeated mice were randomly selected from 15 mice). Data are presented as means ± SEM. ***C***, Paired *t* test and Pearson correlation coefficients. ns, not significant. Detailed results of the statistical tests are described in Extended Data Figure 2-1.

To identify the brain regions that show altered activity following social interaction between the control group and the defeated group, we examined the expression of c-Fos, a marker of neuronal excitation. From day 26, i.e., 18 d after the last defeat session and 2 d after the second series of behavioral tests, each mouse was exposed to an unfamiliar ICR mouse for 5 min and killed 2 h later for c-Fos immunostaining. To efficiently count the number of c-Fos-positive cells in various brain regions in multiple individuals, we established a method to comprehensively extract and count c-Fos-derived signals based on semi-automated image processing. For extracting and quantifying the c-Fos signals obtained by immunostaining, three procedures were applied. In step 1, to extract the c-Fos signals, background noise was reduced from the microscopic images and signals that passed the thresholds for size and brightness were extracted (see Materials and Methods for details). In step 2, to select the brain regions of interest, the microscopic images were overlapped with the best-matching section of the mouse brain atlas (Franklin and Paxinos, 2007), and the brain region of interest was outlined based on the brain atlas. In step 3, for each region of interest, those signals that were extracted as c-Fos signals in step 1 and that lay within the region of interest outlined in step 2 were counted to calculate the number of c-Fos signals per square millimeter in that region. To evaluate the validity of this method, we focused on one particular brain region, the PAG (Fig. 2*A*). First, we compared the region outlined as the PAG with this method (PAG_auto) with the actual PAG region that was manually outlined by a researcher blind to the results of the semi-automated analyses (PAG_manual). The precision of the PAG outlining, i.e., the ratio of the area overlapping between PAG_auto and PAG_manual (PAG_auto∩PAG_manual) to the area of PAG_auto was, on average, >90% (Fig. 2*B*). The sensitivity of PAG outlining, i.e., the ratio of the area of (PAG_auto∩PAG_manual) to the area of PAG_manual was also, on average, >80% (Fig. 2*B*). Next, we compared the results of the c-Fos signal density within the PAG quantified with our semi-automated method with that quantified manually by a researcher blind to the results of the semi-automated analyses. The quantification results did not differ significantly between the two methods for either the control or defeated groups (Fig. 2*C*). Furthermore, we analyzed the overlap between the set of signals extracted as c-Fos signals by the semi-automated method (c-Fos_PAG_auto) and those extracted manually as c-Fos signals (c-Fos_PAG_manual). Both the precision, i.e., the ratio of the number of signals detected in both methods (c-Fos_PAG_auto∩c-Fos_PAG_manual) to the number of signals in c-Fos_PAG_auto, and the sensitivity, i.e., the ratio of the number of signals in (c-Fos_PAG_auto∩c-Fos_PAG_manual) to the number of signals in c-Fos_PAG_manual were ∼80% (Fig. 2*D*).

10.1523/ENEURO.0068-22.2022.f2-1 Extended Data Figure 2-1 Detailed results of the statistical analyses in Figure 2. Download Figure 2-1, XLS file.

### Several brain regions exhibited altered activity between the control and defeated mice

**Figure 3.**
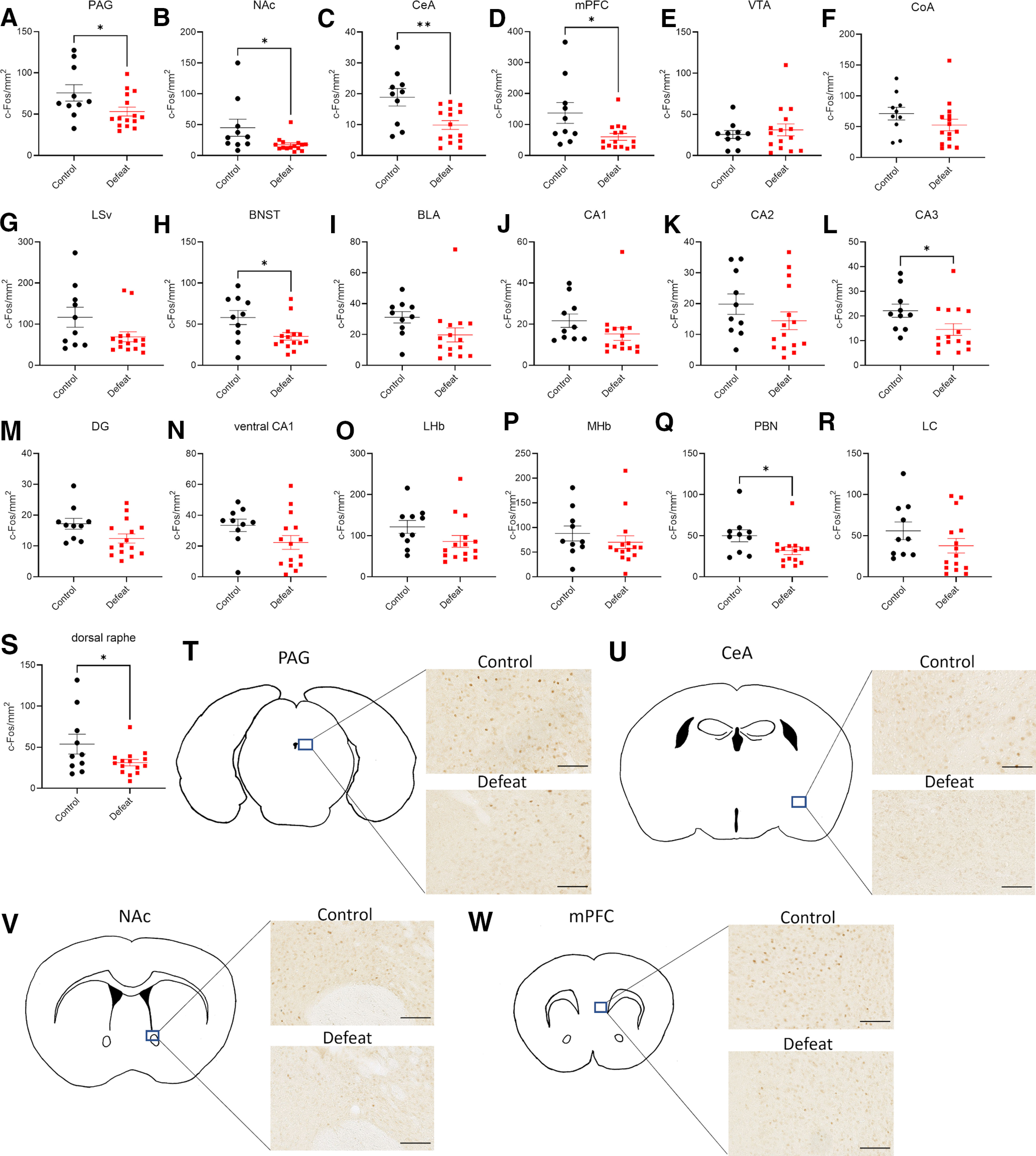
Identification of brain regions that exhibit differential activity between the control and defeated mice following social interaction. ***A–S***, Comparison of c-Fos signal densities between control and defeated mice in various brain regions. PAG, periaqueductal gray; NAc, nucleus accumbens; CeA, central amygdala; mPFC, medial prefrontal cortex; VTA, ventral tegmental area; CoA, cortical amygdala; LSv, lateral septal nucleus ventral part; BNST, bed nucleus of the stria terminalis; BLA, basolateral amygdala; DG, dentate gyrus; LHb, lateral habenula; MHb, medial habenula; PBN, parabrachial nucleus; LC, locus coeruleus. Control: *n* = 10, defeat: *n* = 15, data are presented as means ± SEM, *p < 0.05, **p < 0.01, unpaired *t* test. Detailed results of the statistical tests are described in Extended Data Figure 3-1. ***T–W***, Representative images of c-Fos signals in the PAG (***T***), CeA (***U***), NAc (***V***), and mPFC (***W***) of control and defeated mice. Scale bar: 0.05 mm.

Using the semi-automated method described above, we quantified the c-Fos signal density in various brain regions. We selected 19 candidate regions that are reported to show altered activity immediately after a single defeat session (Numa et al., 2019) or to be related to depression, anxiety, pain, or memory (for review, see Krishnan and Nestler, 2008). Defeated mice had decreased c-Fos signal densities in the PAG, NAc, CeA, mPFC, BNST, CA3, PBN, and the dorsal raphe (Fig. 3*A–D*,*H*,*L*,*Q*,*S*) compared with control mice.

10.1523/ENEURO.0068-22.2022.f3-1 Extended Data Figure 3-1 Detailed results of the statistical analyses in Figure 3. Download Figure 3-1, XLS file.

### Correlation in activity among several brain regions emerged in defeated mice

**Figure 4.**
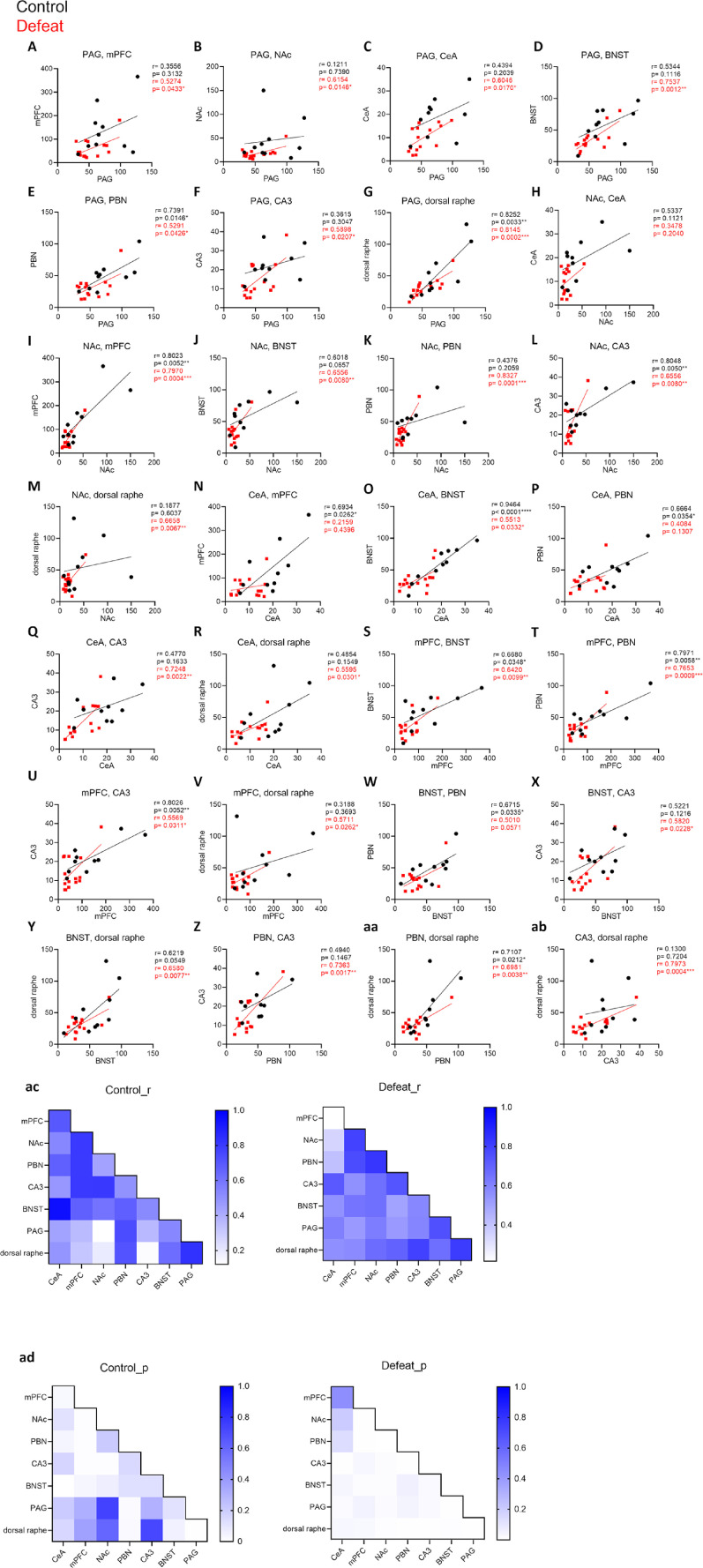
Repeated SDS leads to the emergence of an activity correlation between various brain regions. ***A–ab***, Scatter plots showing correlations in c-Fos signal densities among pairs of brain regions that showed altered activity between control and defeated mice in Figure 3. ***ac***, ***ad***, Heatmaps showing the overview of the r and p values of Pearson correlation coefficients. Control: *n* = 10, defeat: *n* = 15, *p < 0.05, **p < 0.01, ***p < 0.001, ****p < 0.0001, Pearson correlation coefficients. Detailed results of the statistical tests are described in Extended Data Figure 4-1.

To investigate whether the activity of one brain region is linked to that of other regions, we calculated the correlations of c-Fos signal densities among brain regions whose activity was altered between control and defeated mice (Fig. 4). In control mice, significant positive correlations were found in 12 pairs, which, except for NAc to CA3 and mPFC to PBN, are known to contain monosynaptic connections (Fig. 4*E*,*G*,*I*,*L*,*N–P*,*S–U*,*W*,*aa*,*ac*,*ad*; Stezhka and Lovick, 1997; Flavin et al., 2014; Lee et al., 2014; Gungor et al., 2015; Rajasethupathy et al., 2015; Cai et al., 2018; Sun et al., 2019; Chiang et al., 2020; Glover et al., 2020; Lin et al., 2020). In the defeated mice, significant positive correlations were found in almost all pairs of brain regions, implying that the functional connectivity among these brain regions was potentiated by chronic SDS (Fig. 4*A–G*,*I–M*,*O*,*Q–V*,*X*,*ad*). Notably, in contrast to most other pairs of brain regions, significant positive correlations were found between the CeA and mPFC in the control mice, but this positive correlation seemed to be abolished in the defeated mice (Fig. 4*N*). Thus, chronic SDS might lead to a functional dissociation between the CeA and mPFC, brain areas that are involved in coping with stress (Ventura-Silva et al., 2013; Seo et al., 2017).

10.1523/ENEURO.0068-22.2022.f4-1 Extended Data Figure 4-1 Detailed results of the statistical analyses in Figure 4. Download Figure 4-1, XLS file.

### Hierarchical cluster analysis of subpopulations of defeated-mice based on c-Fos signal densities

**Figure 5.**
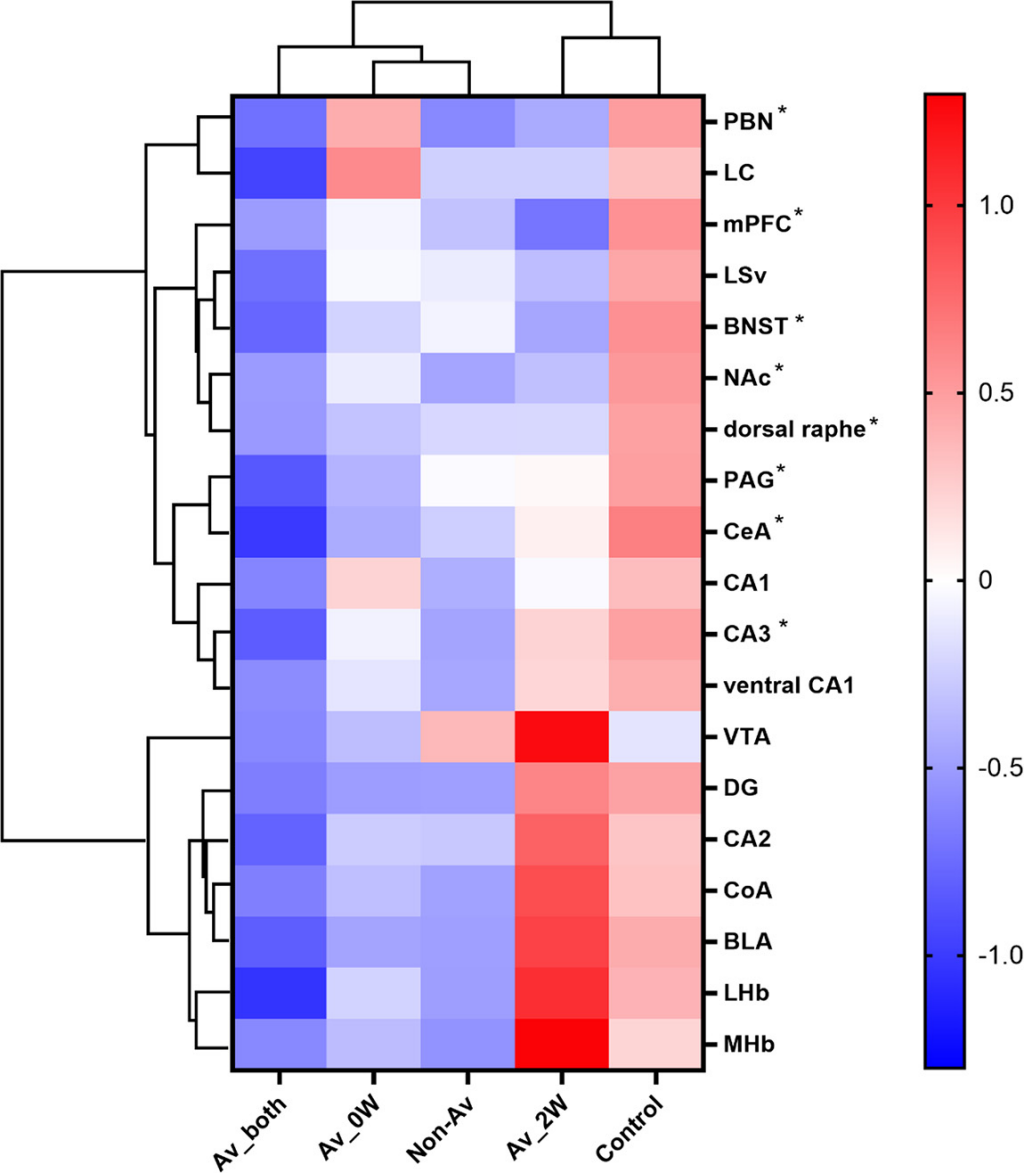
Hierarchical cluster analysis of subpopulations of defeated mice based on c-Fos expression following social interaction 18 d after the repeated SDS. Results of two-way hierarchical clustering analysis are shown together with heatmap visualization of normalized c-Fos signal densities. Av_both indicates mice that were avoidant mice in both SIT_0W and SIT_2W. Av_0W and Av_2W indicate mice that were avoidant in either SIT_0W or SIT_2W, respectively. Non-Av indicates mice that were nonavoidant in both SIT_0W and SIT_2W; * shows the brain regions that showed altered activity between control and defeated mice in Figure 3.

The defeated mice can be separated into four subpopulations based on the results of SIT, i.e., whether they were avoidant or nonavoidant either in SIT_0W or in SIT_2W. We next analyzed the characteristics of each subpopulation in terms of the c-Fos signal distribution following social interaction 18 d from the repeated SDS period. c-Fos signal densities in each of the 19 brain regions were normalized and used for hierarchical cluster analysis (Fig. 5). Consistent with the results of comparison between defeated mice and control mice (Fig. 3), subpopulations of defeated mice generally exhibited low c-Fos signal densities overall, and this trend was especially apparent in mice that were avoidant mice in both SIT_0W and SIT_2W (Av_both; Fig. 5). However, mice that were avoidant only in SIT_2W (Av_2W) were distinct from the other subpopulations in that it contrastingly exhibited high c-Fos signal densities in several brain regions (Fig. 5).

### c-Fos signal density in the LHb differed between Av_both mice and Av_2W mice

**Figure 6.**
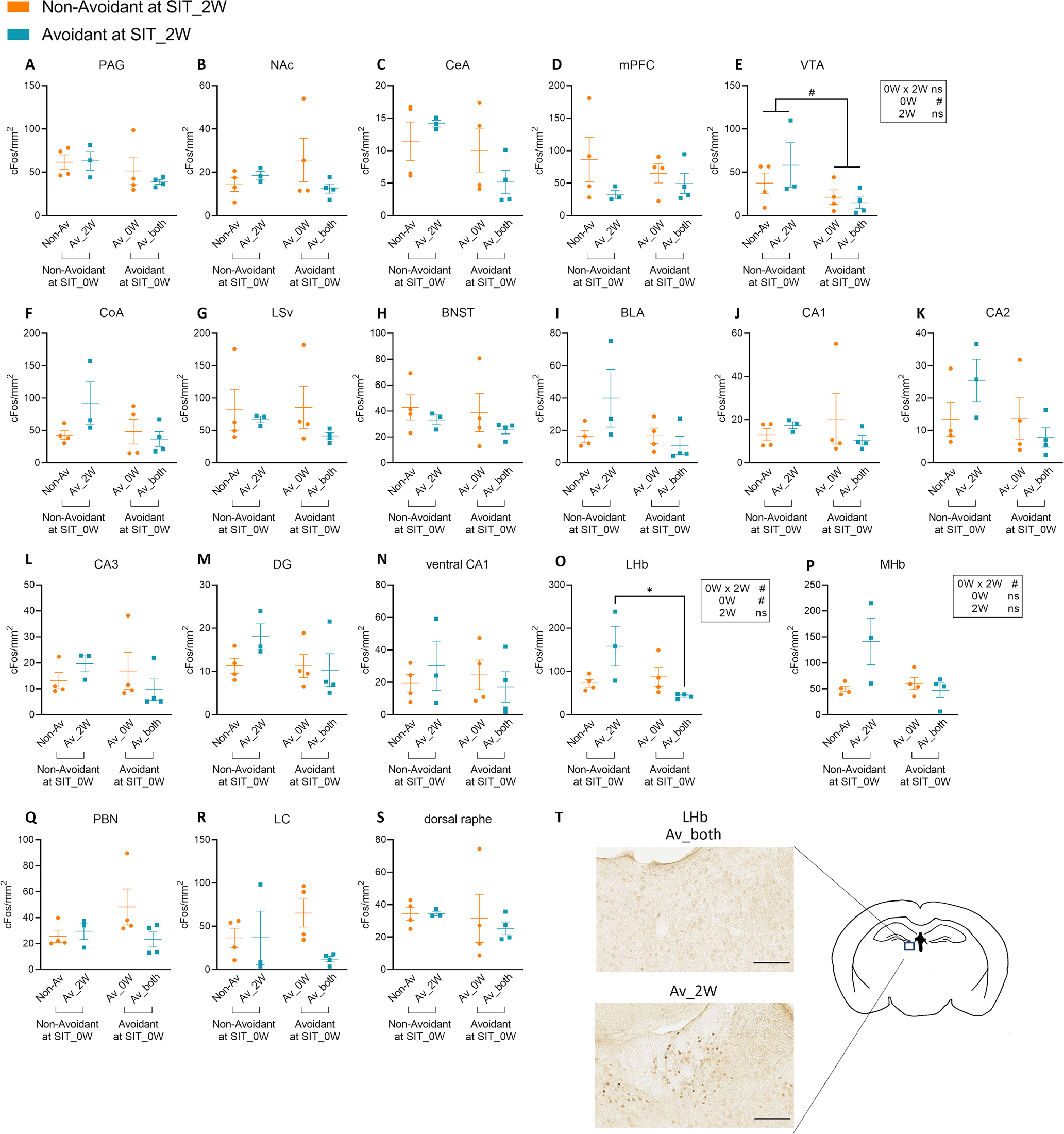
Identification of brain regions that showed altered c-Fos signal densities among subpopulations of defeated mice following social interaction 18 d after the repeated SDS. ***A–S***, Comparison of c-Fos signal densities among the subpopulations of defeated mice defined in Figure 5. Av_both: *n* = 4, Av_0W: *n* = 4, Av_2W: *n* = 3, Non-Av: *n* = 4, data are presented as means ± SEM, */#*p* < 0.05, two-way ANOVA followed by multiple comparison tests with Bonferroni correction. Detailed results of the statistical tests are described in Extended Data Figure 6-1. ***T***, Representative images of c-Fos signals in the LHb of Av_both and Av_2w mice. Scale bar: 0.05 mm.

Lastly, we directly compared the c-Fos signal densities in each of the 19 brain regions among the subpopulations of defeated mice following social interaction 18 d from the repeated SDS period (Fig. 6). As a result, c-Fos signal densities in the LHb were higher in Av_2W mice compared with Av_both mice (Fig. 6*O*). A similar trend was also observed in the MHb (Fig. 6*P*). These results further support that Av_2W mice and Av_both mice show distinct patterns of brain activity during the encounter with ICR mice despite that both mice were avoidant near the timing of c-Fos analyses. In addition, in the VTA, the c-Fos signal densities were lower in mice that were avoidant in the SIT_0W compared with mice that were nonavoidant in the SIT_0W (Fig. 6*E*). Thus, the brain activity state immediately after the repeated SDS that affected the behavior of the defeated mice during the SIT_0W might somehow also affect the response of VTA neurons during the social encounter two weeks later, although the results of the SIT_2W were not relevant to the results of the SIT_0W.

10.1523/ENEURO.0068-22.2022.f6-1 Extended Data Figure 6-1 Detailed results of the statistical analyses in Figure 6. Download Figure 6-1, XLS file.

## Discussion

Many rodent studies have focused on the effects of acute or chronic stress immediately after the end of the stress exposure period, but the long-term effects of prior stress on the stress-induced phenotypes are less well understood. In the present study, we examined the behavioral phenotypes of BALB/c mice that underwent repeated SDS, both immediately and two weeks after the end of the stress exposure period. Immediately after repeated SDS, BALB/c mice exhibited largely decreased body weight and increased social avoidance in the SIT, while anxiety levels assessed by the EPM were unaffected, results that are consistent with previous studies (Laine et al., 2018; Yamagishi et al., 2019; Ishikawa et al., 2021). We further demonstrated that BALB/c mice exposed to repeated SDS exhibited increased social avoidance and concomitant decreased social interest even two weeks after the end of the stress exposure period. Moreover, repeated SDS lead to increased anxiety-like behavior that was evident two weeks after, but not immediately after, the end of the stress exposure period. Finally, neuronal activity during social interaction more than two weeks after the end of the stress exposure period was analyzed using a semi-automated c-Fos extraction and quantitation method, which might provide clues to understanding the long-term effects of repeated SDS on behavior.

Regarding the drastic body weight decrease observed from day 6 in the BALB/c mice, previous studies reported that C57BL/6J mice that underwent repeated SDS exhibited increased body weight (Razzoli et al., 2011; Goto et al., 2014). This body weight gain in defeated C57BL/6J mice is associated with increased intake of food and water (Goto et al., 2014). In contrast, defeated BALB/c mice do not alter the amount of food intake compared with control mice, but instead decrease the feed efficiency (total body weight gain/total food intake; Razzoli et al., 2011). Our results provide further support that repeated SDS has differential effects on BALB/c mice and C57BL/6J mice.

Our results suggest that BALB/c mice that underwent repeated SDS developed anxiety-like behavior at some time point during the post-SDS period. Development of anxiety during the poststress period is also observed in NIH Swiss mice following chronic restraint stress (Chotiwat and Harris, 2006). Together, these findings point to the importance of addressing the long-term effects of chronic stress.

To efficiently compare c-Fos expression between defeated mice and control mice, we developed a semi-automated c-Fos extraction and quantitation method. A similar method was previously applied to analyze c-Fos expression in rats (Bourgeois et al., 2021). Our current results further support the effectiveness of using such semi-automated approaches for quantifying and comparing c-Fos expression densities in multiple brain regions across multiple individuals.

Our approach of comparing c-Fos expression between defeated mice and control mice a long time after the end of exposure to repeated SDS allowed us to address the long-term effects of repeated SDS on brain activity and to exclude the more direct effects of contact with the aggressor mouse, such as injury. Using our semi-automated method, we identified brain regions that exhibited altered brain activity during social interaction. In defeated mice, the c-Fos signal density was decreased in the PAG, NAc, CeA, mPFC, BNST, CA3, PBN, and dorsal raphe relative to control mice. Notably, c-Fos signal densities in many of these regions are rather increased in C57BL/6 mice following a single SDS (Numa et al., 2019). It is possible that repeated SDS leads to a decrease in the excitability of brain regions that are activated by a single SDS, which might underlie the behavioral changes caused by repeated SDS. The results are consistent with reduced activity of the NAc during reward processing in depressed patients (Redlich et al., 2015). In addition, atrophy of the dendrites of excitatory neurons in the mPFC (Shinohara et al., 2018), reduced neurotransmission in the ventrolateral PAG (Ho et al., 2018), and reduced dendritic spines in the CA3 (Qiao et al., 2014) are associated with rodent models of depression. Interestingly, while c-Fos expression in the defeated mice was decreased in many brain regions, the correlation of c-Fos expression between multiple brain regions was increased. These brain regions are deeply related to the processing of anxiety, fear, and pain, and are interconnected (Gonçalves et al., 2009; Kamiyama et al., 2011; Padilla-Coreano et al., 2016; Ahrens et al., 2018; Cheriyan and Sheets, 2018; Liu et al., 2020; for review, see Waselus et al., 2011; Leuner and Shors, 2013; Ong et al., 2019; Price and Duman, 2020). The increased correlation in c-Fos expression may imply that, while the excitability of neurons within each brain region is decreased in the defeated mice, the connections between the brain regions are potentiated. One exception was the correlation between the mPFC and CeA; c-Fos expression was strongly correlated in the control mice but no correlation was detected in the defeated mice. Thus, the connections between mPFC and CeA might be depotentiated by repeated SDS. The connection from the mPFC to the amygdala is attenuated in children with autism spectrum disorder (Li et al., 2021). In mice, activation of the dorsal part of the mPFC to the BLA projection, or inhibition of the ventral part of the mPFC to the BLA projection impairs social behavior (Huang et al., 2020). In our results, we also observed a trend toward a reduced correlation of c-Fos expression between the BLA and mPFC in the defeated mice compared with control mice (control mice: *r* = 0.6296, *p* = 0.0511; defeated mice: *r* = 0.0421, *p* = 0.8817). Thus, a weakening of the connection between the amygdala and mPFC might be involved in increased social avoidance in the defeated mice.

Defeated BALB/c mice segregated into non-avoidant mice and avoidant mice, consistent with previous studies (Laine et al., 2018; Ishikawa et al., 2021). In general, BALB/c mice show higher stress susceptibility compared with C57BL/6 mice in terms of hormonal or gene expression levels (Flint and Tinkle, 2001; Malki et al., 2015; Tsuchimine et al., 2020). Yet, about half of the defeated BALB/c mice were nonavoidant. In our study, comparison of the results of the SIT_0W and SIT_2W revealed that whether each defeated mouse is an avoidant mouse or a nonavoidant mouse is not a fixed trait, although the overall ratio of the two subpopulations was stable. This is in contrast to previous studies using C57BL/6J mice, in which levels of social interaction just 24 h from the end of SDS and after four weeks are strongly correlated (Berton et al., 2006). Currently, it is unclear whether the difference is because of differences in the strain or differences in the experimental design. At least in the case of BALB/c mice, it might not be appropriate to categorize avoidant or nonavoidant mice as “resilient” or “susceptible” individuals. Further studies are required to clarify the factors that underlie the avoidant or nonavoidant phenotype. To obtain some clues in this regard, we divided the defeated mice into four subpopulations based on the combined results of SIT_0W and SIT_2W and compared the patterns of c-Fos signal densities among these subpopulations. Interestingly, although cautious interpretation is necessary considering that the sample sizes of each subpopulation were small, the results of the hierarchical cluster analysis implied that mice that were avoidant only at later periods after SDS (Av_2W) might be distinct from mice that were avoidant in both SIT_0W and SIT_2W (Av_both) in terms of brain activity following social interaction, although both subpopulations were similarly avoidant near the timing of c-Fos analyses. Moreover, direct comparison of c-Fos signal densities revealed that Av_2W mice showed higher levels of c-Fos signal density in the LHb compared with Av_both mice, and a similar trend was observed in the MHb. Likely because of the high variability of c-Fos signal densities in the LHb among the subpopulations, when all subpopulations were combined and compared with the control mice, there was no significant difference (Fig. 3*O*). Neurons in LHb and MHb are excited by aversive stimuli (Ressler et al., 2002; Matsumoto and Hikosaka, 2007; Amo et al., 2014) and are involved in coordinating behavioral responses to that stimuli (Agetsuma et al., 2010; Yamaguchi et al., 2013; Lecca et al., 2017, 2020). In addition, long-term perturbation of LHb or MHb leads to the expression of depression-like behavior or increased anxiety (Yang et al., 2008; Yamaguchi et al., 2013; Cui et al., 2014). In case of Av_2W mice, the activation of neurons in LHb and MHb during the social interaction might underlie the avoidant response. The contrastingly low c-Fos expression in the LHb and MHb of Av_both mice seems more difficult to interpret. Av_both mice exhibited low c-Fos signal densities across all 19 brain regions (Fig. 5). There might be a possibility that the encounter with the ICR mice or the subsequent behavioral response triggered a unique brain state with low neuronal activity in these mice. Further circuit genetic analyses may lead to understanding how the same SDS protocol leads to individual differences in social behavior in the subsequent periods.

We also found that c-Fos signal density in the VTA was lower in mice avoidant in the SIT_0W compared with mice nonavoidant in the SIT_0W, whereas there was no difference between avoidant and nonavoidant mice based on the SIT_2W results. VTA dopaminergic neurons comprise distinct subpopulations that are activated by either reward or aversive stimuli (Lammel et al., 2012). Subpopulations of VTA dopaminergic neurons also play opposing roles in behavioral modifications following repeated SDS in C57BL/6J mice, i.e., those projecting to the NAc promote susceptibility, whereas those projecting to the mPFC promote resilience via the dopamine D1 receptor (Chaudhury et al., 2013; Shinohara et al., 2018). In the case of BALB/c mice, VTA dopaminergic neurons may play critical roles in determining the levels of social avoidance immediately after repeated SDS exposure, whereas other brain regions are involved at later periods.

In summary, we found that BALB/c mice exposed to repeated SDS develop anxiety-like behavior at some time point during the post-SDS period. Moreover, under our protocol, we found that the level of social avoidance in the BALB/c mice exposed to repeated SDS was not fixed and either increased or decreased depending on the individual. These findings point to the importance of understanding changes in brain functions that occur several weeks after the end of exposure to stress. To this end, as a starting point, we identified multiple brain regions in which activity was altered during social interaction more than two weeks after mice were exposed to repeated SDS by using our semi-automated c-Fos extraction and quantitation method. Our experimental design allowed us to exclude c-Fos expression caused by direct physical contact with the aggressor mice. These findings will contribute to a better understanding of how chronic stress causes changes in brain functions during the poststress period.

## Synthesis

Reviewing Editor: Bong-Kiun Kaang, Seoul National University Decisions are customarily a result of the Reviewing Editor and the peer reviewers coming together and discussing their recommendations until a consensus is reached. When revisions are invited, a fact-based synthesis statement explaining their decision and outlining what is needed to prepare a revision will be listed below. The following reviewer(s) agreed to reveal their identity: Miyata Shingo.

The manuscript is interesting but needs to fully address the concerns that are raised by the reviewers stated below before publication.

Reviewer 1:

The authors analyzed long-term behavioral and neuronal effects of repeated social defeat stress in BALB/c mice with higher stress susceptibility than C57BL/6 mice. They found that the level of social avoidance was not fixed over the two weeks after the end of stress. Their semi-automated method for c-Fos expression analysis found that social defeat stress decreased c-Fos expression in many brain areas, whereas the correlation between two of them, except the medial prefrontal cortex and central amygdala, increased. Two weeks after the stress, c-Fos expression in the VTA was negatively correlated to the level of social avoidance immediately (0 weeks) after the stress, thus two weeks before, rather than at the time of analysis. Also, the mice that became avoidant from non-avoidant over the two weeks after the stress showed a different pattern of c-Fos expression higher in a subset of brain areas than those that were avoidant immediately after the stress or remained non-avoidant over the two weeks.

The aim of this research is important to understand chronic stress-induced neuropsychiatric disturbance. The experiments were adequately conducted and analyzed. The findings contain new information meaningful for neuroscientists interested in stress biology. However, there are several points to be addressed to substantiate the conclusions fully.

Major points

1. The authors contrasted their findings with BALB/c mice to previous results from other labs with C57BL/6 mice regarding the stability of social avoidance over the two weeks after the stress. However, the experimental conditions of repeated social defeat stress could be slightly different across studies, such as how many times social defeat stress was given, how the mice were maintained between stress exposure, and how the aggressor mice were chosen. These differences could affect the stability of social avoidance. Thus, the authors should add the experiments with C57BL/6 mice with the same protocol they used for BALB/c mice or de-emphasize their statement about the strain difference.

2. The analyses of c-Fos expression at two weeks after the stress shown in Figures 5 and 6 are somewhat confusing as the same mice were grouped in several ways. In Figure 5, the authors divided the defeated mice into avoidant and non-avoidant groups based on the level of social avoidance immediately (0 weeks) or two weeks after the stress. In Figure 6, the authors divided the same mice into four groups based on the combination of the levels of social avoidance at the two time points. It would be less confusing if the authors showed the same four groups in Figure 5 and analyzed them with two-way ANOVA. The authors need such a statistical analysis to prove Figure 6's argument that the mice that became avoidant from non-avoidant over the two weeks were different from the other mice as the appearance of the heatmap could be affected by outliers.

Minor points

1. This reviewer assumes that Figures 5 and 6 show the results of c-Fos expression two weeks after the stress. However, the authors did not mention it. They should clarify it in both the main text and the figure legends.

2. The authors should show representative images of c-Fos immunostaining for the claims of Figures 3 and 5.

Reviewer 1:

Firstly, the authors showed that defeated BALB/c mice gradually developed anxiety-like behavior after 2 weeks of 7 days repeated SDS. And, the behavior of social avoidance was not fixed in each individual BALB/c mouse over these 2 weeks. Next, the authors developed semi-automated c-Fos extraction and quantification method. The authors further show the correlation between c-Fos expression in VTA and social avoidance behavior immediately after, but not 2 weeks after repeated SDS for BALB/c mice.

Thus, this work appears novel and very interesting and has the potential to impact our understanding mechanisms of the long-term effects of chronic stress on brain function in BALB/c mice. There are, however, some shortcomings that should be reconsidered. These primary concerns are detailed below.

1) The authors investigate the SIT and EPM behaviors 2days after, and 2 weeks after repeated SDS for BALB/c mice. I could not find any comment in this manuscript that this schedule was valid. The authors should more clearly explain in this manuscript why you set this schedule instead of other potentials. Did the authors already check other time settings?

2) There is no data of c-Fos immunostaining image for validation of semi-automated c-Fos extraction and quantification method in Figure 2. Furthermore, in Supplement Figures, the authors should indicate representative c-Fos immunostaining images at several brain regions (NAc, CeA, mPFC, etc) of control and defeated BALB/c mice.

3) On line352-355, the authors state “... repeated SDS gradually developed anxiety-like behavior during the post-SDS period. A gradual increase in anxiety during the post-stress period...” How did the authors find this anxiety-like behavior gradually developed during the post-SDS period?

4) In Figure 6, the authors showed the data of hierarchical cluster analysis of c-Fos expression brain regions. I can understand the data of ‘Av-both’ very low densities of the c-Fos signal at almost all these brain regions. However, why did ‘Av_0W’ and ‘Non_Av’ data indicate almost the same densities of the c-Fos signal at these brain regions?

5) In Figure 6, the authors showed the densities of high c-Fos signal in VTA, hippocampus (DG and CA2), amygdala (CoA and BLA), and habenula nuclei (LHb and MHb) of only ‘Av_2W’ mice brain. Furthermore, almost these brain regions did not show the activity alteration between control and defeat BALB/c mice. Why? Please explain in the Discussion section.
